# The potential role of the Asian bush mosquito *Aedes japonicus* as spillover vector for West Nile virus in the Netherlands

**DOI:** 10.1186/s13071-024-06279-5

**Published:** 2024-06-17

**Authors:** Charlotte Linthout, Afonso Dimas Martins, Mariken de Wit, Clara Delecroix, Sandra R. Abbo, Gorben P. Pijlman, Constantianus J. M. Koenraadt

**Affiliations:** 1 Department of Entomology, Wageningen University & Research, Wageningen, the Netherlands; 2 Department of Population Health Sciences, Faculty of Veterinary Medicine, Utrecht University, Utrecht, the Netherlands; 3 Quantitative Veterinary Epidemiology, Wageningen University & Research, Wageningen, the Netherlands; 4 Department of Environmental Sciences, Wageningen University & Research, Wageningen, the Netherlands; 5 Department of Virology, Wageningen University & Research, Wageningen, the Netherlands

**Keywords:** *Aedes japonicus*, Spillover, Vector, Vector competence, West Nile virus, the Netherlands

## Abstract

**Background:**

In recent years the Asian bush mosquito *Aedes japonicus* has invaded Europe, including the Netherlands. This species is a known vector for a range of arboviruses, possibly including West Nile virus (WNV). As WNV emerged in the Netherlands in 2020, it is important to investigate the vectorial capacity of mosquito species present in the Netherlands to estimate the risk of future outbreaks and further spread of the virus. Therefore, this study evaluates the potential role of *Ae. japonicus* in WNV transmission and spillover from birds to dead-end hosts in the Netherlands.

**Methods:**

We conducted human landing collections in allotment gardens (Lelystad, the Netherlands) in June, August and September 2021 to study the diurnal and seasonal host-seeking behaviour of *Ae. japonicus*. Furthermore, their host preference in relation to birds using live chicken-baited traps was investigated. Vector competence of field-collected *Ae. japonicus* mosquitoes for two isolates of WNV at two different temperatures was determined. Based on the data generated from these studies, we developed a Susceptible-Exposed-Infectious-Recovered (SEIR) model to calculate the risk of WNV spillover from birds to humans via *Ae. japonicus*, under the condition that the virus is introduced and circulates in an enzootic cycle in a given area.

**Results:**

Our results show that *Ae. japonicus* mosquitoes are actively host seeking throughout the day, with peaks in activity in the morning and evening. Their abundance in August was higher than in June and September. For the host-preference experiment, we documented a small number of mosquitoes feeding on birds: only six blood-fed females were caught over 4 full days of sampling. Finally, our vector competence experiments with *Ae. japonicus* compared to its natural vector *Culex pipiens* showed a higher infection and transmission rate when infected with a local, Dutch, WNV isolate compared to a Greek isolate of the virus. Interestingly, we also found a small number of infected *Cx. pipiens* males with virus-positive leg and saliva samples.

**Conclusions:**

Combining the field and laboratory derived data, our model predicts that *Ae. japonicus* could act as a spillover vector for WNV and could be responsible for a high initial invasion risk of WNV when present in large numbers.

**Graphical Abstract:**

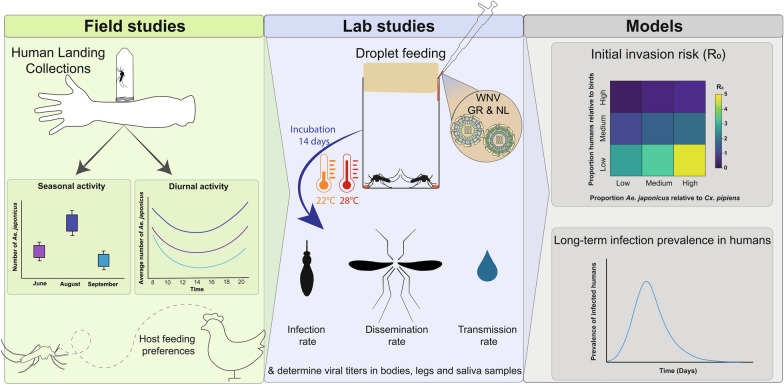

**Supplementary Information:**

The online version contains supplementary material available at 10.1186/s13071-024-06279-5.

## Background

West Nile virus (WNV; family *Flaviviridae*, genus *Flavivirus*) is a widespread arthropod-borne virus, transmitted by mosquitoes in an enzootic cycle. It circulates between mosquitoes and birds, whereas humans and horses are considered dead-end hosts [1]. The virus was first isolated in 1937 from a woman with fever in the West Nile region in Uganda [2]; from there, it likely spread via infected birds through the African continent and led to occasional epidemics in Europe and East Asia. Since the introduction of WNV lineage 1 in North America in 1999 [3], it rapidly spread over the USA towards Canada [4] and to Central and South America [5]. In Europe, WNV had been circulating for a while before causing an outbreak in Greece in 2010 [6]. In 2020, WNV lineage 2 was detected for the first time in the Netherlands in a common whitethroat (*Curruca communis*) and later also in pools of *Culex pipiens* mosquitoes in the same region [7]. Six human cases of West Nile neuroinvasive disease or West Nile fever were also reported in that year [8].

Native and invasive mosquito species, such as *Aedes japonicus*, can be potential vectors for mosquito-borne viruses, including WNV. In the Netherlands, invasive *Aedes japonicus* mosquitoes were first detected during a routine surveillance in 2013 in the municipality of Lelystad [9] and have been able to become established in and around that area since then. The *Ae. japonicus* mosquito is indigenous to Japan, Korea, Taiwan, eastern China and Russia [10] and has expanded its territory because of increased travel and trade. This container-dwelling mosquito species colonises both human-made and natural habitats. It is an opportunistic feeder, as it feeds on both avian and mammalian hosts [11, 12]; therefore, it could potentially act as a spillover vector for arboviruses to dead-end hosts such as humans and horses.

The ability of mosquitoes to transmit pathogens (vector competence) in combination with several other factors, such as temperature, mosquito species, biting behaviour, available hosts and pathogen genetics, determines the vectorial capacity of a certain vector-pathogen system [13]. Temperature has an effect on the vector competence of mosquitoes for a range of arboviruses [14], which is likely linked to temperature-induced changes in the extrinsic incubation period (EIP) and the longevity and feeding rate of mosquitoes [15, 16]. Additionally, the genetic composition of the virus isolate affects vector competence in local mosquito species [17].

Previous studies have investigated the vector competence of *Ae. japonicus* mosquitoes for WNV, Zika virus and Usutu virus in Europe [18–21]. However, these studies yielded conflicting results. Furthermore, they did not account for other aspects of vectorial capacity and ecology of this mosquito species. The current research therefore not only investigates the vector competence of invasive *Ae. japonicus* mosquitoes at different temperatures for two different WNV isolates (both lineage 2, from Greece 2010 and the Netherlands 2021), but also combines most factors influencing the vectorial capacity. We investigated the biting behaviour of *Ae. japonicus* mosquitoes in allotment gardens near Lelystad during the summer of 2021 by performing human landing collections (HLC). Furthermore, we studied the seasonal biting activity of this mosquito species as well as the biting rate on chickens to obtain information on the host preference of this mosquito. In the biosafety level 3 laboratory of Wageningen University, we experimentally investigated the vector competence of *Ae. japonicus* mosquitoes for WNV. Lastly, Susceptible-Exposed-Infectious-Recovered (SEIR) models have been described for WNV based on previously collected data [22, 23]. However, these models have not yet been used to model the potential contribution of *Ae. japonicus* mosquitoes in the spillover of WNV. Therefore, we created a SEIR model which integrates data from the field and the laboratory in order to evaluate the potential role of *Ae. japonicus* and *Cx. pipiens* in the spillover of WNV from birds to humans.

## Methods

### Study area

*Aedes japonicus* populations are present in and around allotment gardens in the municipality of Lelystad (52˚31′42.6"N, 5˚28′00.6"E), the Netherlands. Three locations within and around these allotment gardens were therefore selected for our studies. These locations were between 250 and 395 m apart and were selected based on previous data showing high *Ae. japonicus* activity for these locations. Human landing collections (HLC) were performed, and oviposition traps were set out to collect mosquito eggs.

### Field collection and identification of mosquitoes

HLCs were conducted to study the diurnal and seasonal host-seeking dynamics of *Ae. japonicus* mosquitoes. Over 3 days in June (22, 23 and 24 June 2021), August (2, 3 and 5 August 2021) and September (27, 29 and 30 September 2021), six volunteers were divided into three groups of two. From 8 a.m. until sunset, each group visited an allocated spot every hour for 15 min. Each hour teams were mixed to account for bias in relative attractiveness of the volunteers. After exposing their arms and legs and being present in the area for 5 min, the volunteers used a 50-ml tube to collect all individual mosquitoes landing on them for the next 15 min. After each sampling moment, the collected mosquitoes were pooled per volunteer and per time slot in 1.5-ml Eppendorf tubes and were put on ice for transport. After transport to Wageningen University, the collected mosquitoes were identified to species level using the identification key of Becker et al. [24].

During August and September 2021, *Ae. japonicus* eggs and larvae were collected in and around the allotment gardens of Lelystad, as previously described by Abbo et al. [21]. In brief, oviposition traps, consisting of a black plastic flowerpot (Elho, Tilburg, the Netherlands), were placed in shaded areas and close to trees. These traps contained approximately 3.5 l rainwater, a handful of hay and a floating Styrofoam block of approximately 5*5*2 cm (l*b*h). The Styrofoam blocks, intended for egg laying by mosquitoes, were collected and replaced every 2 weeks. Furthermore, a landing net (10 cm * 7.5 cm) was used to collect larvae from rain barrels in private allotment gardens after permission was given by the landowner.

### *Aedes japonicus* and *Culex pipiens* rearing

The collected *Ae. japonicus* eggs were transported to the secure insect rearing facilities of Wageningen University. Styrofoam blocks with eggs were placed in plastic buckets with 1.5 l demineralised water and a drop of Liquifry No. 1 (Interpet Ltd., Dorking, UK) at 23 °C, 60% RH and a 16:8 light:dark period. Hatched larvae were fed with Tetramin baby fish food (Tetra, Melle, Germany) every 2–3 days. Pupae were collected in a cup and transferred to Bugdorm cages (30 × 30 × 30 cm; MegaView Science Co., Ltd., Taichung, Taiwan) to emerge. The emerged adults received 6% glucose solution ad libitum as a food source and were used for follow-up studies on vector competence.

*Culex pipiens pipiens* mosquitoes (F9 progeny of field-collected mosquitoes) were used as a control in the vector competence studies. During the summer of 2020, several oviposition traps consisting of 4 l tap water and 1 l hay infusion were placed on public and private land (with permission) close to chicken coops across Wageningen, the Netherlands. The hay infusion was prepared by incubating a handful of hay in 1 l tap water at room temperature in dark and anaerobic conditions for 7 days. Every 3 days, egg rafts were collected from the oviposition traps and individually transported to the insectary at Wageningen University (23 °C, 60% relative humidity and a 16:8 light:dark period). After the eggs hatched, 10 larvae originating from one egg raft were identified to biotype level as described by Vogels et al. [25]. The remaining larvae from the egg raft with biotype *pipiens* were further reared as described by Vogels et al. [26]. Adult mosquitoes were provided with 6% glucose solution ad libitum and chicken blood was offered to the females for reproduction.

### Chicken-baited traps in the field

**Fig. 1:**
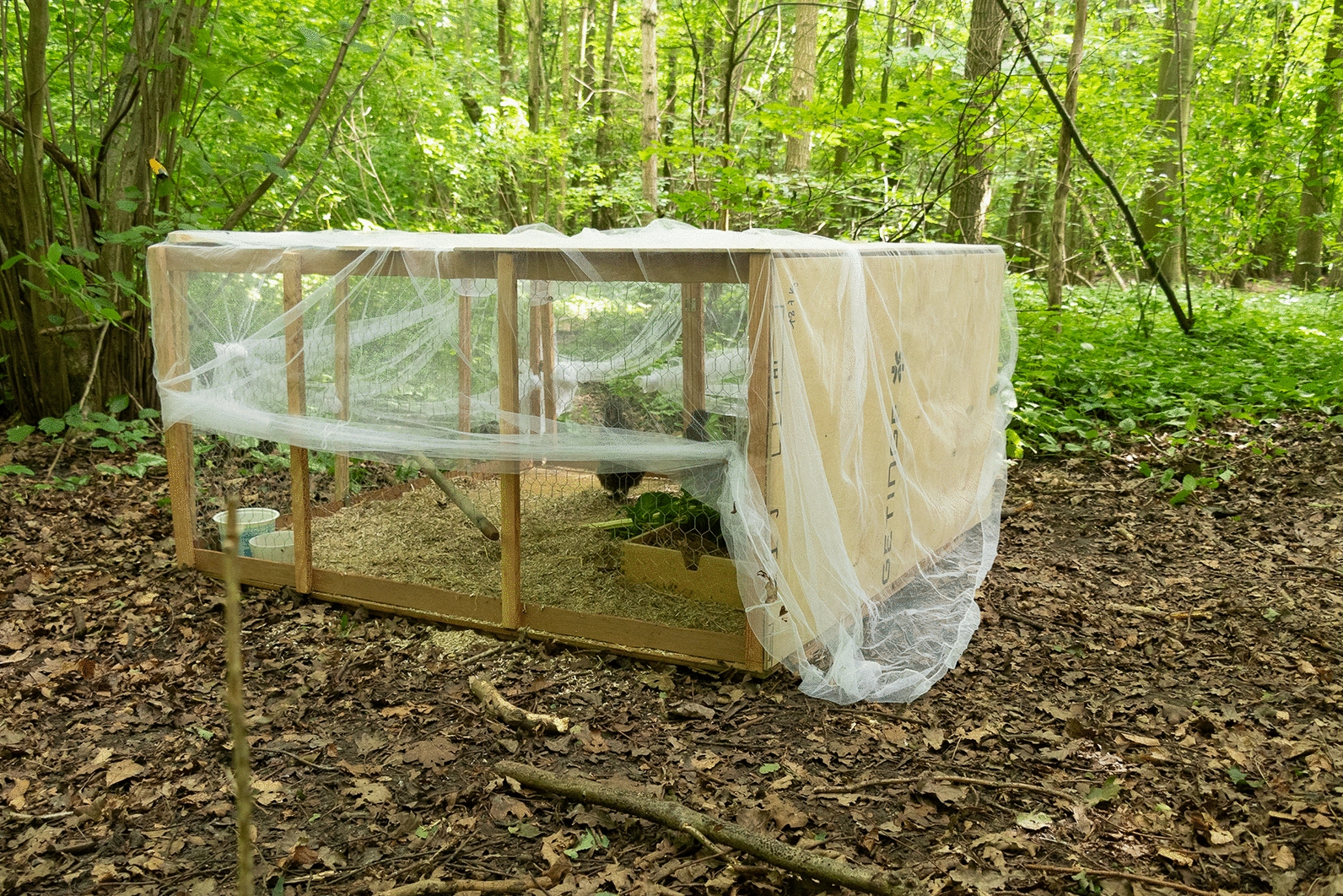
Chicken baited trap with lifted mosquito net

As a model for evaluating the attractiveness of *Ae. japonicus* to birds, we used caged Wugu-ji chickens (*Gallus gallus domesticus* Brisson) in our experimental work. For this purpose, a live chicken-baited trap (145 × 145 × 75 cm) was placed at a location with a high abundance of *Ae. japonicus*. On each of 4 days in August and September (18 August 2021, 1 September 2021, 15 September 2021 and 28 September 2021), when HLCs were not taking place, a pair of Wugu-ji chickens were placed in the trap from sunrise to sunset. A mosquito net, which was draped over the trap, allowed mosquitoes to enter the trap via the lower part of the sides (Fig. 1). Each hour, the mosquito net was dropped down and the chicken-baited trap was checked for the presence of (blood-fed) mosquitoes. If present, mosquitoes were collected using a mouth aspirator and stored in Eppendorf tubes at − 20 °C for species typing and blood meal analysis.

Use of chickens as bait for mosquito collection was exempted from the Dutch Law on Animal Experiments. Nevertheless, handling and care procedures for the chickens were performed according to the guidelines of the Dutch Law for Animal Experiments.

### West Nile virus

Two lineage 2 West Nile virus stocks were used for mosquito oral inoculation: a passage 2 WNV Greek lineage 2 from 2010 (GenBank accession no. HQ537483.1) and a passage 3 Dutch WNV lineage 2 (unpublished), which was isolated during the WNV outbreak in 2021 in the Netherlands. The Greek isolate originated from a mosquito pool and the Dutch isolate originated from a common chiffchaff (*Phylloscopus collybita*). Both were kindly provided by Erasmus Medical Center (Rotterdam, the Netherlands).

WNV was grown at 37 ℃ on a monolayer of Green monkey kidney Vero E6 cells, cultured with Hepes-buffered Dulbecco’s Modified Eagle Medium (DMEM; Gibco, Carlsbad, CA, USA) and titrated on the same cell line to determine the viral loads in virus-positive samples. This medium was supplemented with 10% fetal bovine serum (FBS; Gibco), Gentamicin (50 μg/ml, Gibco), Fungizone (2.5 μg/ml amphotericin B and 2.1 μg/ml sodium deoxycholate; Gibco) and a combination of penicillin (100 U/ml; Sigma-Aldrich, Saint Louis, MO, USA) and streptomycin (100 μg/ml; Sigma-Aldrich, Zwijndrecht, the Netherlands) (P/S). This medium is hereafter referred to as DMEM Hepes complete. The Vero E6 cells were cultured with DMEM (Gibco) and supplemented as described above and incubated at 37 ℃ and 5% CO_2_.

### Vector competence studies

Four- to 11-day-old *Ae. japonicus* (*n* = 449) and *Cx. pipiens* (*n* = 742) females were used in vector competence studies. Both mosquito species received an infected blood meal with either the Greek or Dutch WNV isolate. The infected blood meal was offered to the mosquitoes via droplet feeding, as previously described by Jansen et al. [27] and Abbo et al. [21]. The droplets consisted of human blood (Sanquin Blood Supply Foundation, Nijmegen, the Netherlands), mixed with 10% FBS, 1.6% fructose and the virus isolates mentioned above. The final virus titre of the blood meals ranged between 8.79 * 10^5^ TCID_50_/ml and 8.66 * 10^6^ TCID_50_/ml (verified by End Point Dilution Assay, EPDA). After 2 to 3 h of blood feeding, mosquitoes were anesthetized with CO_2_ and the fully engorged females were selected. To test the effect of temperature on the vector competence of the mosquitoes, we placed them at 22 or 28 °C in bucket cages with a 16:8 light:dark cycle for a 14-day period. As a food source, 6% glucose solution was provided via cotton wool on top of the bucket during the incubation period.

### Forced salivation assay

Fourteen days post infection, all females were anesthetized using CO_2_. Legs and wings of each female were removed and collected in 1.5-ml SafeSeal Eppendorf tubes containing 5 mm zirconium oxide beads (Next Advance, AverillPark, NY, USA). To collect the saliva of a mosquito, the proboscis was inserted into a 200-μl pipet tip containing 5 μl of a solution of 50% FBS and 25% sugar in tap water. After 45 min, the mosquito body was removed and stored in a 1.5-ml SafeSeal Eppendorf tube containing 5 mm zirconium oxide beads. The individual mosquito saliva samples were resuspended in 55 μl DMEM Hepes complete and stored in 1.5-ml Eppendorf tubes. All individual body, leg and saliva samples were stored at − 80 ℃ until further use in the infectivity assay.

### Infectivity assay

Frozen body and leg samples were homogenised in DMEM Hepes complete using the Bullet Blender Storm (Next Advance, USA), as described by Vogels et al*.* [28]. Thirty µl of each mosquito body, leg or saliva sample was added to each well of a 96-well plate with a 90% confluent monolayer of Vero E6 cells in 60 µl DMEM Hepes complete. After 2 h of incubation at 37 ℃, 100 μl of fresh DMEM Hepes complete medium was added to the wells and the plates were further incubated at 37 ℃ for 6 days. After this incubation period, the wells were scored virus-positive or virus-negative based on the presence of cytopathic effect (CPE). The infection, dissemination and transmission rates were calculated as the number of positive body, leg or saliva samples, respectively, as percentage of the total number of mosquitoes analysed. The transmission efficiency was calculated as the number of positive saliva samples divided by the number of infected bodies for each treatment.

### Virus titration

For the positive body, leg and saliva samples, the viral titres were determined via EPDA on Vero E6 cells. Serial tenfold dilutions (10^–1^ to 10^–9^) of the virus-positive samples were made in DMEM Hepes complete. Vero cells were detached from the surface of a T25 flask and diluted to 5 × 10^5^ cells/ml. These cells were then added in a 1:1 ratio to the virus dilutions. Of this mixture, 10 μl was added to six wells of a 60-well MicroWell plate (Nunc, Roskilde, Denmark). After 6 days, the viral titre expressed as tissue culture infectious dose 50% (TCID_50_) per ml was determined based on CPE observed in the wells.

### Blood meal analysis

To characterise the blood meal source of the blood-fed mosquitoes collected in the chicken-baited trap, the abdomen was first separated from the rest of the mosquito’s body. DNA was then extracted using the DNeasy Blood & Tissue Kit (Qiagen, Hilden, Germany) per the manufacturer’s protocol. For identification of the blood meal source, a previously described PCR [29] with a universal vertebrate primer set, targeting cytochrome B, was performed. For each PCR reaction, MyTaq HS Red Mix (Bioline) was used with the following temperature profile: 1 min at 95 °C, followed by 30 cycles of 15 s at 95 °C, 15 s at 55 °C and 10 s at 72 °C. The fragments were visualized by gel electrophoresis and the PCR products were sequenced (Eurofins Genomics, Ebersberg, Germany). The received sequences were identified using Nucleotide BLAST against the NCBI GenBank database.

### Statistical analysis

One-way analysis of variance (ANOVA) and Tukey HSD were used to compare the differences in the number of *Ae. japonicus* adults collected in June, August and September. Generalised linear models (GLM) with binomial distribution and logit link function were used to test for the effects of replicate, mosquito species, temperature and WNV isolate origin, as well as their two-way interactions (explanatory variables), on the WNV infection, dissemination and transmission rates (dependent variables) of the orally infected mosquitoes. Final models were selected using stepwise backward elimination resulting in different models for the infection, dissemination and transmission rates (Additional file 1: Table S1). Mosquitoes with virus-positive saliva but a virus-negative body (2%) were excluded from the analysis. The effect of WNV isolate on viral titres was tested separately for each mosquito species at each temperature with a Wilcoxon test. All statistical analyses, as well as all figures, were made using R statistical software, packages stats, ggpubr, ggplot2, lme4 and DHARMa, version 4.3.1.

### Modelling spillover risk to humans

#### Model development

To assess the potential invasion and spillover risk of WNV from birds to humans, we developed a SEIR model (adapted from Wonham et al. [22]) that included two vector species, *Cx. pipiens* biotype *pipiens* (*N*_*p*_ = *S*_*p*_ + *E*_*p*_ + *I*_*p*_) and *Ae. japonicus* (*N*_*j*_ = *S*_*j*_ + *E*_*j*_ + *I*_*j*_), and two host species, birds (*N*_*b*_ = *S*_*b*_ + *E*_*b*_ + *I*_*b*_ + *R*_*b*_) and humans (*N*_*h*_ = *S*_*h*_ + *E*_*h*_ + *I*_*h*_ + *R*_*h*_). The equations for this system are:

$${S}_{p}^{{\prime} }={\Delta }_{p}\left(T\right)-{b}_{p}\left(T\right){p}_{bp}\left(T\right){S}_{p}\frac{{I}_{b}}{{N}_{b}+{N}_{h}}-{\mu }_{p}\left(T\right){S}_{p}$$

$${S}_{j}^{{\prime} }={\Delta }_{j}\left(T\right)-{b}_{j}\left(T\right){p}_{bj}\left(T\right){S}_{j}\frac{{I}_{b}}{{N}_{b}+w{N}_{h}}-{\mu }_{j}\left(T\right){S}_{j}$$

$${S}_{b}^{{\prime} }={\Delta }_{b}-{b}_{p}\left(T\right){p}_{pb}\left(T\right){I}_{p}\frac{{S}_{b}}{{N}_{b}+{N}_{h}}-{b}_{j}\left(T\right){p}_{jb}\left(T\right){I}_{j}\frac{{S}_{b}}{{N}_{b}+w{N}_{h}}-{\mu }_{b}{S}_{b}$$

$${S}_{h}^{{\prime} }={\Delta }_{h}-{b}_{p}\left(T\right){p}_{ph}\left(T\right){I}_{p}\frac{{S}_{h}}{{N}_{b}+{N}_{h}}-{b}_{j}\left(T\right){p}_{jh}\left(T\right){I}_{j}\frac{w{S}_{h}}{{N}_{b}+w{N}_{h}}-{\mu }_{h}{S}_{h}$$

$${E}_{p}^{{\prime} }={b}_{p}\left(T\right){p}_{bp}\left(T\right){S}_{p}\frac{{I}_{b}}{{N}_{b}+{N}_{h}}-\left({\mu }_{p}\left(T\right)+{\sigma }_{p}\left(T\right)\right){E}_{p}$$

$${E}_{j}^{{\prime} }={b}_{j}\left(T\right){p}_{bj}\left(T\right){S}_{j}\frac{{I}_{b}}{{N}_{b}+w{N}_{h}}-\left({\mu }_{j}\left(T\right)+{\sigma }_{j}\left(T\right)\right){E}_{j}$$

$${E}_{b}^{{\prime} }=\,{b}_{p}\left(T\right){p}_{pb}\left(T\right){I}_{p}\frac{{S}_{b}}{{N}_{b}+{N}_{h}}-{b}_{j}\left(T\right){p}_{jb}\left(T\right){I}_{j}\frac{{S}_{b}}{{N}_{b}+w{N}_{h}}-\left({\mu }_{b}+{\sigma }_{b}\right){E}_{b}$$

$${E}_{h}^{{\prime} }=\,{b}_{p}\left(T\right){p}_{ph}\left(T\right){I}_{p}\frac{{S}_{h}}{{N}_{b}+{N}_{h}}-{b}_{j}\left(T\right){p}_{jh}\left(T\right){I}_{j}\frac{w{S}_{h}}{{N}_{b}+w{N}_{h}}-\left({\mu }_{h}+{\sigma }_{h}\right){E}_{h}$$

$${I}_{p}^{{\prime} }={\sigma }_{p}\left(T\right){E}_{p}-{\mu }_{p}\left(T\right){I}_{p}$$

$${I}_{j}^{{\prime} }={\sigma }_{j}\left(T\right){E}_{j}-{\mu }_{j}\left(T\right){I}_{j}$$

$${I}_{b}^{{\prime} }={\sigma }_{b}{E}_{b}-\left({\mu }_{b}+{\alpha }_{b}+{\gamma }_{b}\right){I}_{b}$$

$${I}_{h}^{{\prime} }={\sigma }_{h}{E}_{h}-\left({\mu }_{h}+{\alpha }_{h}+{\gamma }_{h}\right){I}_{h}$$

$${R}_{b}^{{\prime} }={\gamma }_{b}{I}_{b}-{\mu }_{b}{R}_{b}$$

$${R}_{h}{\prime}={\gamma }_{h}{I}_{h}-{\mu }_{h}{R}_{h}$$

We used the data from the vector competence studies to inform the values of the transmission parameters for *Cx. pipiens* and *Ae. japonicus*. Values and definitions of all other parameters are presented in Additional file 2: Table S2 [22, 23, 26, 30–36].

#### General assumption s

The following six assumptions are made in the SEIR model: (i) There is a constant recruitment (birth and migration) rate, Δ_*i*_, for every species, with *i* ∈ {*p, j, b, h*}. The mortality rates are constant for each species and given by *μ*_*i*_. (ii) Transmission is modelled as being dependent on total frequency of host abundance, *N*_*b*_ plus *N*_*h*_. Transmission happens from mosquitoes to birds, and vice versa, and from mosquitoes to humans, but not from humans to mosquitoes. The transmission rates, *β*_*ij*_, are given by the mosquito biting rates, *b*_*p*_ and *b*_*j*_, multiplied by the probability that the virus is transmitted, *p*_*ij*_. Detailed information on the estimation of biting rate and transmission probabilities is provided in Additional file 3: Text S3 [22, 37, 38]. (iii) Mosquitoes become infected after the extrinsic incubation period (EIP), expressed by the inverse of the transition rates from latent to infectious compartments, *σ*_*p*_ and *σ*_*j*_. The same interpretation holds for the hosts, which can only become infectious after the intrinsic incubation period (IIP). (iv) Bird and human hosts are affected by an additional death rate caused by the infection, *α*_*b*_ and *α*_*h*_, and recover from it at rates *γ*_*b*_ and *γ*_*h*_. We assume that the vectors are not affected by the infection and therefore do not experience an additional death rate nor recover from it. (v) *Aedes japonicus* has a feeding preference towards humans relative to the birds if *w* > 1, and no preference exists if *w* = 1 (i.e. bites are taken at random between humans and birds). To reflect a feeding preference of *Ae. japonicus* to humans compared to birds [39], we assume *w* = 5 (i.e. 83% of bites are on humans rather than on birds) in our simulations, using an approach to modelling biting preferences similar to [40]. (vi) Several parameters vary by temperature (*T*); these include mosquito recruitment rate, mosquito death rate, biting rate and extrinsic incubation period.

#### Modelled scenarios

The invasion risk of WNV and the long-term infection prevalence in humans was simulated under 36 different scenarios, reflected by assuming different values for the parameters: (i) two temperature levels (22 and 28 °C) that reflect potential future climate scenarios or different European regions at risk of WNV invasion; (ii) two WNV isolates (Greek, Dutch), to quantify the relative risk of a new isolate being introduced relative to another. The assumption is that the two isolates do not directly interact with each other. They do, however, differ in terms of transmissibility. (iii) There are three relative proportions of *Ae. japonicus* compared to *Cx. pipiens* to compare different ecosystems: 10% *Ae. japonicus* (low), 50% *Ae. japonicus* (medium) and 90% *Ae. japonicus* (high). (iv) There are three relative proportions of humans compared to birds to compare among environments. The three relative proportions used for the hosts are the same as those used for the vectors.

When modelling infection risk, it has been suggested that the vector-to-host ratio is a key determinant for the value of *R*_*0*_ [26]. Therefore, to avoid having the vector-to-host ratio as a confounding factor, it is fixed and the proportion of *Ae. japonicus* is assumed to be the complement of that of *Cx. pipiens* (that is *q*_*j*_ = 1-*q*_*p*_) and the proportion of humans to be the complement of that of birds (*q*_*h*_ = 1-*q*_*b*_).

#### Outcome measures: basic reproduction number and long-term prevalence in the human population

To investigate the initial risk of a WNV outbreak under the 36 different scenarios, two outcome measures were investigated: the basic reproduction number, *R*_*0*_, and the prevalence in humans over a longer term (up to 200 days). R_0_ is derived from the next-generation matrix from the full model, with full derivation available in the Additional file 4: Text S4 [41]. All deterministic numerical simulations were carried out using the deSolve package with the ode function in R version 4.2.0.

#### Elasticity analysis

To study the sensitivity of R_0_ to the choice of parameter values, an elasticity analysis was conducted. Details on the methods and results are presented in Additional file 5: Text S5.

## Results

### Seasonal and diurnal biting behaviour of *Ae. japonicus* around human bait

**Fig. 2:**
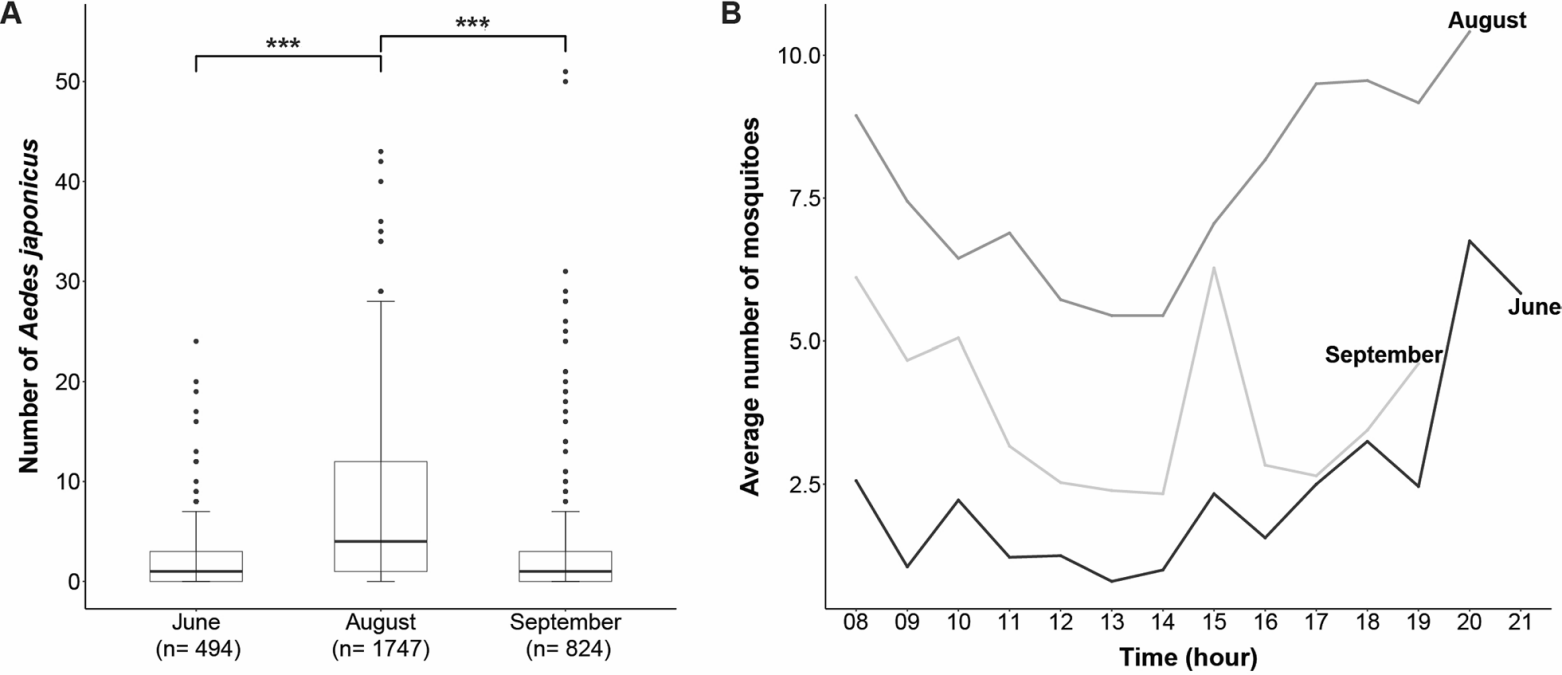
Biting activity of *Aedes japonicus* throughout the season in Lelystad, the Netherlands. Each data point presents the total number of mosquitoes collected per person per 15 min (**A**). Biting activity of *Ae. japonicus* throughout the day in Lelystad, the Netherlands. Lines reflect the average number of collected mosquitoes per person per 15 min (**B**). Asterisks (*) indicate significant differences based on a one-way analysis of variance and Tukey HSD (***P < 0.001)

During 9 sampling days over 3 months, a total of 3065 *Ae. japonicus* mosquitoes were collected via human landing collections. The collections in August were significantly higher than those in June and September (*P* < 0.001, Tukey HSD) (Fig. 2A). On a full day of sampling, volunteers collected relatively more mosquitoes after sunrise and before sunset compared to the rest of the day (Fig. 2B). An unexpected peak in activity was recorded in September at 15:00 h. This peak was the result of a high number of mosquitoes collected by two of the volunteers on one of the sampling days.

### Biting behaviour of *Ae. japonicus* around a live chicken-baited trap

On 4 days in August and September 2021, the live chicken-baited trap was employed and a total of six fully engorged *Ae. japonicus* females were collected in a time frame of 48 sampling hours. Bloodmeal analysis confirmed that five of these six mosquitoes took a blood meal on the chickens. For one sample we could not generate a sufficiently high DNA yield; therefore, no bloodmeal source could be identified. While observing the cage from a distance, we noticed a substantial number of *Ae. japonicus* mosquitoes approaching and entering the cage. However, not all mosquitoes managed to feed on the chickens because of the predation by the chickens on those mosquitoes that entered.

Using the same set-up with human bait, we demonstrated that 48 mosquitoes could be collected within just 1 h. This confirmed that the chicken-baited trap design was effective in attracting and trapping host-seeking *Ae. japonicus*.

### Dutch *Ae. japonicus* mosquitoes can experimentally transmit WNV under constant temperatures in the laboratory

Cohorts of 4–11-day-old *Ae. japonicus* from the field were infected with WNV to investigate their vector competence. As a positive control vector for WNV, *Cx. pipiens* mosquitoes (4–16 days old) were also offered an infectious bloodmeal via droplet feeding.

The droplet feeding rates on the WNV-spiked blood ranged between 40 and 72% among the replicates (average 55%) for *Ae. japonicus* and between 56 and 67% among the replicates (average 62%) for *Cx. pipiens*.

After 14 days of incubation, the presence of virus in the mosquito body, legs and wings, and saliva was determined by infectivity assays on Vero cells.

**Fig. 3:**
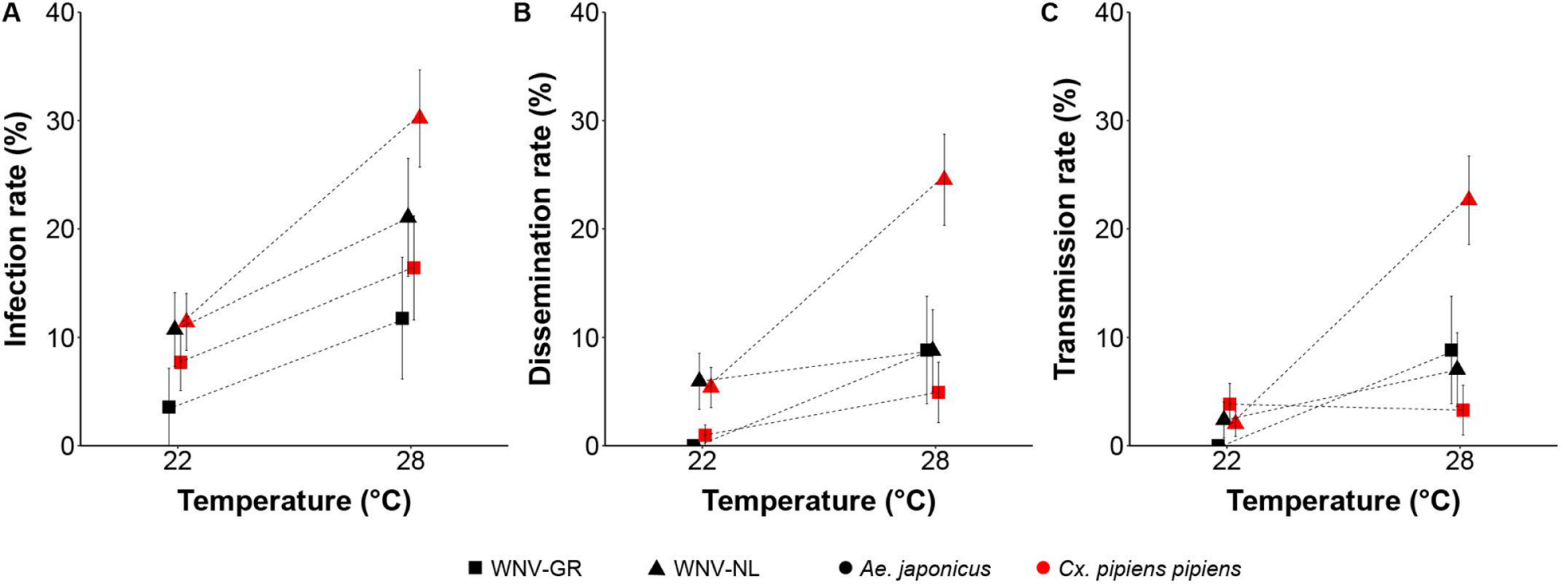
Vector competence of *Aedes japonicus* and *Culex pipiens pipiens* for West Nile virus. Infection (**A**), dissemination (**B**) and transmission (**C**) rates of orally infected *Ae. japonicus* and *Cx. p. pipiens* mosquitoes infected with the Dutch (NL) or Greek (GR) isolate of WNV. Error bars represent the standard error of the mean. Three replicates per data point were performed

GLM analyses of WNV infection rates (Table 1 and Fig. 3A) showed that the numbers of infected mosquitoes with the Dutch WNV isolate were significantly higher (GLM, *χ*^2^ = 7.58, *df* = 1, *P* = 0.006) than the numbers of mosquitoes infected with the Greek WNV isolate. The infection rates of mosquitoes incubated at 28 °C were also significantly higher (GLM, *χ*^2^ = 20.31, *df* = 1, *P* < 0.001) compared to the mosquitoes incubated at 22 °C. Similar results were found for the dissemination rates (Fig. 3B), where a significantly higher disseminated infection rate was observed in mosquitoes when infected with the Dutch isolate than with the Greek isolate (GLM, *χ*^2^ = 15.36, *df* = 1, *P* < 0.001) and when incubated at 28 °C compared to 22 °C (GLM, *χ*^2^ = 23.06, *df* = 1, *P* < 0.001). No significant interaction effects were found among explanatory variables for the model of infection rates (GLM, *χ*^2^ = 2.04, *df* = 1, *P* = 0.15) or for the model of dissemination rates (GLM, *χ*^2^ = 2.39, *df* = 1, *P* = 0.12). (Fig. 3A, B). Finally, the statistical analysis of the transmission rates showed a significant interaction effect (GLM, *χ*^2^ = 8.76, *df* = 2, *P* = 0.01) between WNV isolate and incubation temperature. This indicated that with higher temperatures, mosquitoes infected with the Dutch isolate of the virus showed higher transmission rates than mosquitoes infected with the Greek isolate of the virus, which has lower transmission rates with increasing temperatures (Fig. 3C).

**Table 1:** Vector competence of *Aedes japonicus* and *Culex pipiens pipiens* for WNV, with infection, dissemination and transmission rates, and transmission efficiency

Mosquito species	WNV isolate	Incubation temperature (℃)	Feeding rate (%)	Survival rate (%)	Infection rate (%)	Dissemination rate (%)	Transmission rate (%)	Transmission efficiency (%)
*Ae. japonicus*	GR	22	29/65 (45)	28/29 (97)	1/28 (4)	0/28 (0)	0/28 (0)	0/1 (0)
*Ae. japonicus*	NL	22	85/118 (72)	84/85 (99)	9/84 (11)	5/84 (6)	2/84 (2)	2/9 (22)
*Ae. japonicus*	GR	28	51/128 (40)	34/51 (67)	4/34 (12)	3/34 (9)	3/34 (9)	3/4 (75)
*Ae. japonicus*	NL	28	85/138 (62)	57/85 (67)	12/57 (21)	5/57 (9)	4/57 (7)	4/12 (33)
*Cx. pipiens*	GR	22	105/186 (56)	104/105 (99)	8/104 (8)	1/104 (1)	1/104 (4)	1/8 (13)
*Cx. pipiens*	NL	22	150/ 232 (65)	149/150 (99)	17/149 (11)	8/149 (5)	3/149 (2)	3/17 (18)
*Cx. pipiens*	GR	28	88/131 (67)	61/88 (69)	10/61 (16)	3/61 (5)	2/61 (3)	2/10 (20)
*Cx. pipiens*	NL	28	111/193 (58)	106/111 (95)	32/106 (30)	26/106 (25)	24/106 (23)	24/32 (75)

### Viral titres in virus-positive body and saliva samples

**Fig. 4:**
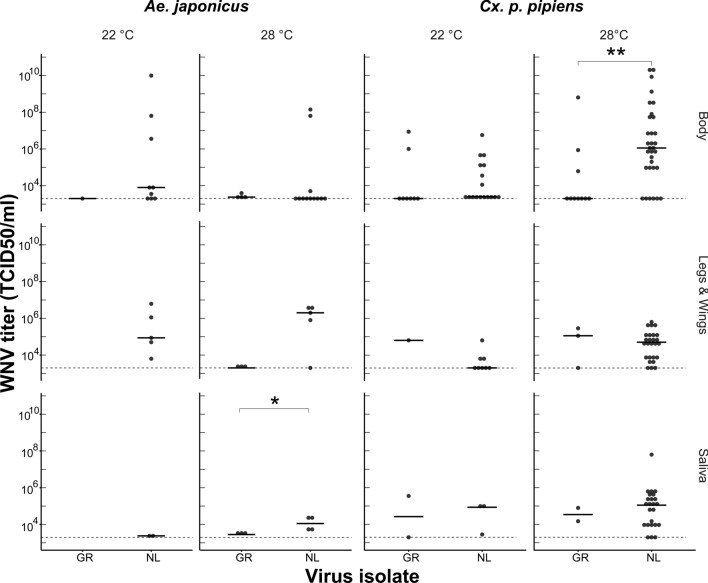
West Nile Virus titres in body, legs and wings and saliva samples of *Aedes japonicus* and *Culex pipiens pipiens* at 22 and 28 °C. Asterisks (*) indicate significant differences (*P < 0.05, **P < 0.01) based on a Wilcoxon test. *GR* Greek WNV isolate, *NL* Dutch WNV isolate, *TCID* Tissue culture infectious dose. Closed horizontal lines show the median viral titres. Dashed lines show the detection limit of the EPDA

We measured viral titres of all mosquitoes with virus-positive body and saliva samples (Fig. 4). The effect of the WNV isolate on the infection dissemination and transmission titres was tested separately for both *Cx. p. pipiens* and *Ae. japonicus* mosquitoes at 22 and 28 °C and was only found to be significantly higher at 28 °C in the saliva samples of *Ae. japonicus* (*P* < 0.05) and in the body samples for *Cx. p. pipiens* (*P* < 0.01)*.*

### Positive WNV-infected *Cx. pipiens* males

During droplet feeding, 34 *Cx p. pipiens* and *Ae. japonicus* males were accidentally included and fed on WNV-spiked blood. A total of 24 blood-fed *Cx. pipiens* and two *Ae. japonicus* males were still alive after incubation at 28 ℃ for 14 days. After the salivation assay and infectivity assay, WNV was detected in the bodies of a total of 4 out of 24 *Cx. pipiens* males (16.7%). Two of these were infected with the Dutch isolate and two with the Greek isolate. Furthermore, three males had a disseminated infection (two with the Dutch isolate and one with the Greek isolate) and two males even had WNV in their saliva (one with the Dutch isolate and one with the Greek isolate) (Table 2). The two *Ae. japonicus* males were negative.

**Table 2:** Summary of male mosquitoes used in the experiment, with infection, dissemination and transmission rates

Mosquito species	WNV isolate	Incubation temperature (℃)	Blood-fed males	Alive after incubation (%)	Infection rate (%)	Dissemination rate (%)	Transmission rate (%)
*Aedes japonicus*	NL	28	4	2/4 (50)	0/2 (0)	0/2 (0)	0/2 (0)
*Culex pipiens*	GR	28	17	12/17 (71)	2/12 (17)	1/12 (8)	1/12 (8)
*Culex pipiens*	NL	28	13	12/13 (92)	2/12 (17)	2/12 (17)	1/12 (8)

### Initial invasion risk

We simulated 36 scenarios based on two temperature values, two different WNV isolates, three *Ae. japonicus-Cx. pipiens* ratios and three human-bird ratios. The scenarios have different, fixed parameters related to several life history elements such as vector competence and birth rates (see Additional file 2: Table S2 for the detailed differences and sources). We compared these scenarios regarding the initial invasion risk of WNV expressed as the basic reproduction number *R*_*0*_.

**Fig. 5:**
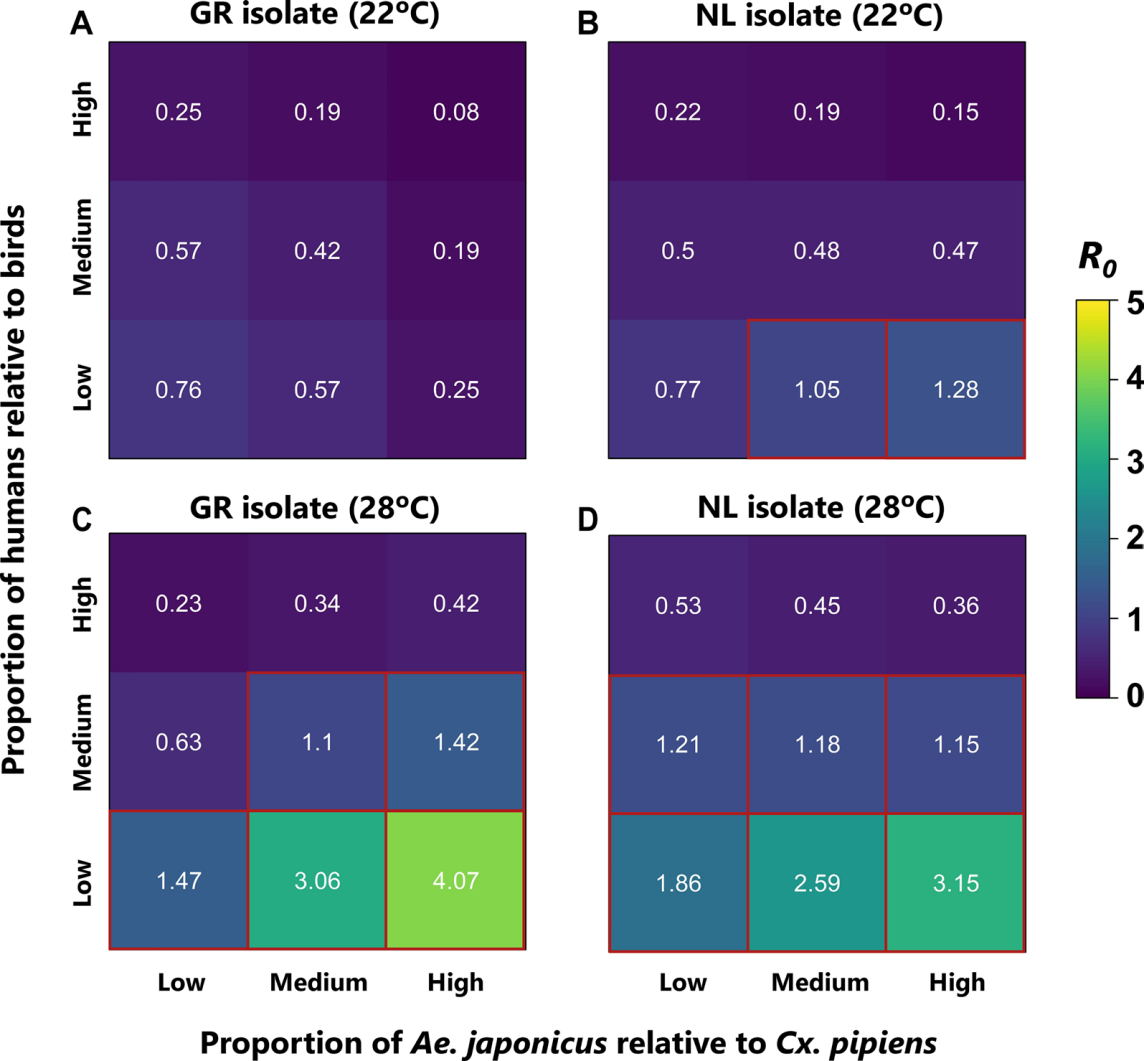
Exploration of relative invasion risk of WNV under different scenarios. Two temperature levels were considered, 22 °C (**A** and **B**) and 28 °C (**C** and **D**), and two isolates of different origin were compared, from Greece (GR, **A** and **C**) and from the Netherlands (NL, **B** and **D**). Along the x-axis, we assumed three levels for the proportion of *Aedes japonicus*, q_j_, relative to *Culex pipiens*, *q*_p_ (with *q*_p_ = 1-*q*_j_) and on the y-axis, three levels for the proportion of humans, *q*_h_, relative to birds, *q*_b_ (with *q*_b_ = 1-*q*_h_). All other parameters were fixed on their point estimates provided in Additional file 2: Table S2 and Additional file 3: Text S3. Colour represents the expected number of infected individuals caused by one infected individual in an otherwise susceptible population, *R*_0_ (low numbers of expected new cases in dark blue, high numbers of expected new cases in yellow). Red squares indicate *R*_0_ > 1 scenarios

Higher temperature was the most impactful driver of WNV invasion risk for both isolates (Fig. 5). In six of the nine scenarios, the Dutch isolate seemed to lead to higher *R*_*0*_ values at 22 ℃ compared to the Greek isolate. In fact, the Greek isolate at 22 ℃ never led to *R*_*0*_ values > 1 but showed higher *R*_*0*_ values at 28 ℃ compared to the Dutch isolate. Additionally, a high proportion of *Ae. japonicus* relative to *Cx. pipiens* led to a higher initial invasion risk. Overall, the combination of high temperature and low proportion of humans relative to birds proved to be the highest risk setting.

### Long-term infection prevalence in humans

To explore the potential effect of WNV invasion on human infection risk, we simulated possible epidemics for the conditions where the invasion risk was highest (temperature 28 °C and host population consisting of 10% humans and 90% birds). We ran 100 simulations of this system with the initial populations of susceptible vectors and hosts set to the disease-free levels expected by the model and the initial population of infected *Cx. pipiens* and *Ae. japonicus*, *I*_*p*_(0) and *I*_*j*_(0) being uniformly sampled from the interval [0, 10].

**Fig. 6:**
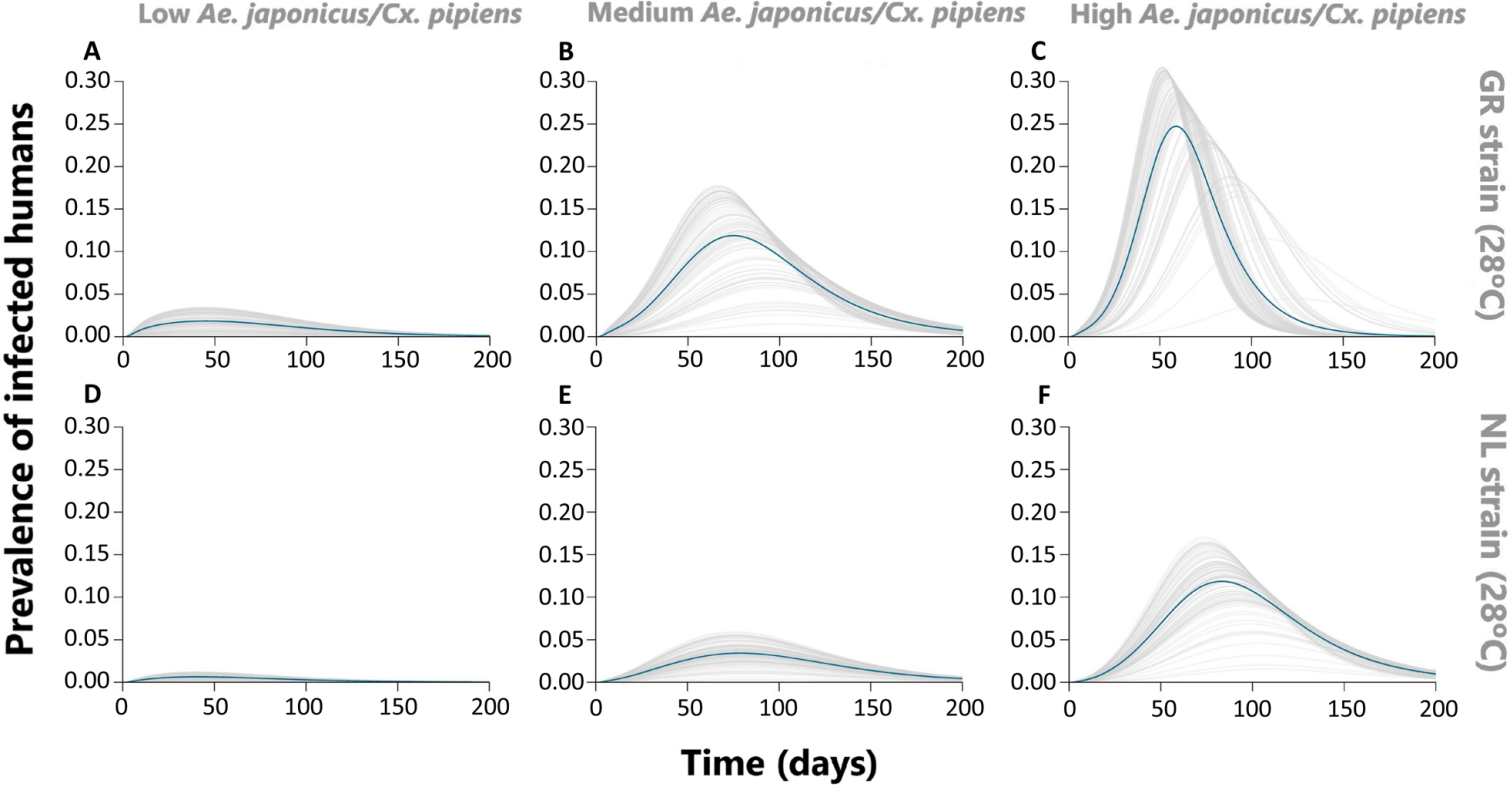
Modelled prevalence of infected humans over time after introduction of the WNV GR isolate (**A**–**C**) or the WNV NL isolate (**D**–**F**) at 28 ºC. All scenarios correspond to a low human ratio compared to birds (10% human, 90% bird) and three ratios of *Aedes japonicus* compared to *Culex pipiens*: low (**A** and **D**), medium (**B** and **E**) and high (**C** and **F**). Each grey line represents a different simulation of infected *Cx. pipiens* and *Ae. japonicus* numbers (samples from the interval [0,10]), at the start of the epidemic. The blue line represents the average of these different simulations

Simulations of those settings that showed the highest invasion risk indicated that when the abundance of *Ae. japonicus* was low compared to *Cx. pipiens* abundance, the prevalence of infected humans was also low (Fig. 6). However, as *Ae. japonicus* became more abundant, prevalence of infected humans increased, and peak prevalence was reached sooner after the first introduction. This effect was strongest for the Greek WNV isolate.

## Discussion

Thus far, WNV has only been detected in *Ae. japonicus* mosquitoes collected from field sites in Tompkins County, New York [42] and Pennsylvania [12]. Although there are no reports of WNV-positive *Ae. japonicus* mosquitoes collected in European countries, previous studies have indicated that this mosquito species is competent for WNV as well as the closely related Usutu virus in the laboratory under realistic European temperature regimes [18, 19, 21, 43, 44]. These findings, combined with the opportunistic feeding behaviour of the species and high biting intensity as confirmed in the present study [11, 12], suggest a possible role of *Ae. japonicus* in the transmission cycle of WNV in Europe.

Here we demonstrate that field-collected *Ae. japonicus* mosquitoes from the Netherlands can experimentally transmit WNV lineage 2 under realistic summer temperatures. Ingestion of the Dutch isolate of WNV lineage 2 resulted in higher infection rates than the Greek isolate of the virus. Similarly, we observed higher infection rates in *Cx. pipiens* for which we previously confirmed that it is a highly competent species for WNV [28, 45–47]. Furthermore, the viral titres of the body, leg and saliva samples were higher for the mosquitoes infected with the Dutch WNV isolate compared to the Greek WNV isolate, irrespective of mosquito species and incubation temperature. These results highlight the importance of using a geographically relevant mosquito species in combination with a geographically relevant virus isolate to assess the risk of mosquito-borne virus circulation in a specific area. Moreover, previous studies have shown the importance of interactions between viral isolates, viral titres and geographically distinct vector populations [45, 48, 49] as well as adaptive evolution of a virus [17] and colonization in the laboratory [50–52] and possibly the mosquito virome [53].

As vector competence is only one component of vectorial capacity [54], we also investigated the biting behaviour of *Ae. japonicus* in its natural environment, in this case allotment gardens near Lelystad, the Netherlands. Sampling from sunrise to sunset in June, August and September proved that *Ae. japonicus* mosquitoes are actively host-seeking throughout the day, with peaks in activity during dawn and dusk. These results are in line with the host-seeking behaviour of other *Aedes* species, such as *Aedes albopictus* and *Ae. aegypti* [55–58]. Furthermore, we investigated the biting behaviour of *Ae. japonicus* mosquitoes throughout the season and our findings are in line with those from Krupa et al*.* [59] and Früh et al*.* [60]. These studies indicate that the peak collection of *Ae. japonicus* eggs in Germany and France occurs in late July and early August. Besides the host-seeking behaviour towards humans, it is important to investigate the host seeking behaviour towards birds in order to assess the mosquito’s potential to act as a bridge vector for WNV. Our results confirmed findings of earlier studies that show the opportunistic feeding behaviour of *Ae. japonicus* mosquitoes on humans, birds, horses, deer and other mammals [11, 12].

To assess the impact of our findings on potential WNV invasion risk, we carried out two modelling exercises that (i) estimated the basic reproduction number and (ii) predicted the temporal development of an epidemic under various scenarios. Our modelling analysis suggests that the WNV invasion risk can be high, especially in scenarios where ambient temperature is high, where birds are highly abundant relative to humans, and where *Ae. japonicus* is highly abundant relative to *Cx. pipiens*. The Dutch WNV isolate seemed to lead to higher *R*_*0*_ values at 22 ℃ compared to the Greek isolate, whereas the Greek isolate showed higher *R*_*0*_ values at 28 ℃. Given the potential of *Ae. japonicus* to act as a spillover vector, we also specifically studied prevalence in humans across several scenarios. This showed that if the Greek isolate of the virus were introduced in a naïve and relatively warm (28 °C) environment, the prevalence of infected humans over time would increase with increasing abundance of *Ae. japonicus*. This would imply that the introduction of the Greek isolate into favourable environmental circumstances could pose a significant risk for human health.

Interestingly, we also observed that male *Ae. japonicus* could develop a disseminated WNV infection. A small number of the males (7.7%) even carried the virus in their saliva. Even though male mosquitoes do not naturally take blood meals, these results indicate a potential role of male mosquitoes in the transmission cycle of WNV, for example by venereal transmission after infection by vertical transmission via the mother mosquito. Vertical transmission of WNV has been demonstrated in *Culex* mosquitoes [61–63], and flavivirus venereal transmission has been demonstrated for Zika virus [64] and dengue virus [65]. Although the likelihood of infected males contributing to a WNV outbreak is low, further studies could investigate venereal transmission of WNV by *Cx. pipiens* mosquitoes to understand the role of infected males in WNV dynamics during an outbreak.

## Conclusions

We conclude that *Ae. japonicus* mosquitoes may contribute to the spillover of WNV from birds to humans in the Netherlands despite their relatively low vector competence at 22 °C (realistic summer temperatures for the Netherlands). The WNV invasion risk is highest in settings with high temperatures, high numbers of birds compared to humans and high numbers of *Ae. japonicus* compared to *Cx. pipiens*. Given the WNV outbreak in the Netherlands in 2020 [7], these new findings raise the importance of awareness of the circulation of WNV in an area where *Ae. japonicus* mosquitoes are highly abundant.

### Supplementary Information

**Additional file 1: Table S1.** Overview on explanatory variables included in the final GLM for the investigated dependent variables.

**Additional file 2: Table S2.** Parameter estimates used for R0 calculation and simulation analysis. All rates are per day.

**Additional file 3: Text S3. **Estimation of biting rate and transmission probabilities.

**Additional file 4: Text S4 **Derivation of the basic reproduction number.

**Additional file 5: Text and Figure S5 **Elasticity of the basic reproduction number.

## Data Availability

The data described in this article can be freely and openly accessed at 10.17026/LS/0DK22V.
