# Supplementary material for: The potential role of the Asian bush mosquito Aedes japonicus as spillover vector for West Nile virus in the Netherlands
Source: Parasit Vectors. 2024 Jun 17;17:262. doi: 10.1186/s13071-024-06279-5 (PMC11181672; doi:10.1186/s13071-024-06279-5)
Supplement: Supplementary file 1 — Additional file 1: Table S1. Overview on explanatory variables included in the final GLM for the investigated dependent variables. [file 13071_2024_6279_MOESM1_ESM.docx]

**Supplementary file1:**

**Table S1**: Overview on explanatory variables included in the final GLM for the investigated dependent variables

|  |  | Dependent variables | | |
| --- | --- | --- | --- | --- |
|  |  | Infection rate | Dissemination rate | Transmission rate |
| Explanatory variables | Replicate | / | / | / |
|  | WNV isolate origin | x | x | x |
|  | Mosquito species | x | x | x |
|  | Incubation temperature | x | x | x |
|  | Incubation temperature : WNV isolate origin | / | / | x |
|  | Incubation temperature : Mosquito species | / | / | / |
|  | Mosquito species : WNV isolate origin | / | / | / |
