# Supplementary material for: The potential role of the Asian bush mosquito Aedes japonicus as spillover vector for West Nile virus in the Netherlands
Source: Parasit Vectors. 2024 Jun 17;17:262. doi: 10.1186/s13071-024-06279-5 (PMC11181672; doi:10.1186/s13071-024-06279-5)
Supplement: Supplementary file 2 — Additional file 2: Table S2. Parameter estimates used for R0 calculation and simulation analysis. All rates are per day. [file 13071_2024_6279_MOESM2_ESM.docx]

**Supplementary File 2:**

**Table S2**: Parameter estimates used for R_0_ calculation and simulation analysis. All rates are per day

|  | Description | Value | Source |
| --- | --- | --- | --- |
|  | General parameters |  |  |
| *Δ_b_* | Bird recruitment rate | 0.5 | [1] |
| *μ_b_* | Bird death rate | 0.001 | [1] |
| *Δ_h_* | Human recruitment rate | 0.000055 | [2] |
| *μ_h_* | Human death rate | 3.91x10^-5^ | [3] |
| *σ_b_* | Transition rate from latent to infected in birds (1/IIP) | 0.555 | [4] |
| *σ_h_* | Transition rate from latent to infected in humans (1/IIP) | 0.25 | [5] |
| *γ_b_* | Bird recovery rate | 0.31 | [4] |
| *γ_h_* | Human recovery rate | 0.0714 | [1] |
| *α_b_* | Additional WNV death rate in birds | 0.2 | [4] |
| *α_h_* | Additional WNV death rate in humans | 5x10^-7^ | [1] |
| *w* | *Ae. japonicus* preference to humans | 5 | Assumed |
| *Δ_p_* | *Cx. pipiens* recruitment rate at 22ºC | 0.39525 | [6] |
| *Δ_j_* | *Ae. japonicus* recruitment rate at 22ºC | 4.047 | [7] |
| *μ_p_* | *Cx. pipiens* death rate at 22ºC | 0.1677 | [6] |
| *μ_j_* | *Ae. japonicus* death rate at 22ºC | 0.037 | [7] |
| *b_p_* | *Cx. pipiens* biting rate at 22ºC | 0.17 | [8] |
| *b_j_* | *Ae. japonicus* biting rate at 22ºC | 0.16 | Estimated |
| *σ_p_* | Transition rate from latent to infected in *Cx. pipiens* (1/EIP) at 22ºC | 0.11 | [8] |
| *σ_j_* | Transition rate from latent to infected in *Ae. japonicus* (1/EIP) at 22ºC | 0.09* | Assumed |
| *Δ_p_* | *Cx. pipiens* recruitment rate at 28ºC | 0.465 | [6] |
| *Δ_j_* | *Ae. japonicus* recruitment rate at 28ºC | 8.294 | [7] |
| *μ_p_* | *Cx. pipiens* death rate at 28ºC | 0.3537 | [6] |
| *μ_j_* | *Ae. japonicus* death rate at 28ºC | 0.028 | [7] |
| *b_p_* | *Cx. pipiens* biting rate at 28ºC | 0.2 | [8] |
| *b_j_* | *Ae. japonicus* biting rate at 28ºC | 0.25 | Estimated |
| *σ_p_* | Transition rate from latent to infected in *Cx. pipiens* (1/EIP) at 28ºC | 0.25 | [8] |
| *σ_j_* | Transition rate from latent to infected in *Ae. japonicus* (1/EIP) at 28ºC | 0.09 | [9] |
|  | Greek isolate |  |  |
| *p_pb_* | Transmission probability *Cx. pipiens* to bird at 22ºC | 0.88 | [10] |
| *p_jb_* | Transmission probability *Ae. japonicus* to bird at 22ºC | 0.88 | Assumed |
| *p_bp_* | Transmission probability bird to *Cx. pipiens* at 22ºC | 0.048 | Estimated |
| *p_bj_* | Transmission probability bird to *Ae. japonicus* at 22ºC | 0 | Estimated |
| *p_ph_* | Transmission probability *Cx. pipiens* to human at 22ºC | 0.88 | [10] |
| *p_jh_* | Transmission probability *Ae. japonicus* to human at 22ºC | 0.88 | Assumed |
| *p_pb_* | Transmission probability *Cx. pipiens* to bird at 28ºC | 0.88 | [10] |
| *p_jb_* | Transmission probability *Ae. japonicus* to bird at 28ºC | 0.88 | Assumed |
| *p_bp_* | Transmission probability bird to *Cx. pipiens* at 28ºC | 0.031 | Estimated |
| *p_bj_* | Transmission probability bird to *Ae. japonicus* at 28ºC | 0.116 | Estimated |
| *p_ph_* | Transmission probability *Cx. pipiens* to human at 28ºC | 0.88 | [10] |
| *p_jh_* | Transmission probability *Ae. japonicus* to human at 28ºC | 0.88 | Assumed |
|  | Dutch isolate |  |  |
| *p_pb_* | Transmission probability *Cx. pipiens* to bird at 22ºC | 0.88 | [10] |
| *p_jb_* | Transmission probability *Ae. japonicus* to bird at 22ºC | 0.88 | Assumed |
| *p_bp_* | Transmission probability bird to *Cx. pipiens* at 22ºC | 0.024 | Estimated |
| *p_bj_* | Transmission probability bird to *Ae. japonicus* at 22ºC | 0.0256 | Estimated |
| *p_ph_* | Transmission probability *Cx. pipiens* to human at 22ºC | 0.88 | [10] |
| *p_jh_* | Transmission probability *Ae. japonicus* to human at 22ºC | 0.88 | Assumed |
| *p_pb_* | Transmission probability *Cx. pipiens* to bird at 28ºC | 0.88 | [10] |
| *p_jb_* | Transmission probability *Ae. japonicus* to bird at 28ºC | 0.88 | Assumed |
| *p_bp_* | Transmission probability bird to *Cx. pipiens* at 28ºC | 0.237 | Estimated |
| *p_bj_* | Transmission probability bird to *Ae. japonicus* at 28ºC | 0.089 | Estimated |
| *p_ph_* | Transmission probability *Cx. pipiens* to human at 28ºC | 0.88 | [10] |
| *p_jh_* | Transmission probability *Ae. japonicus* to human at 28ºC | 0.88 | Assumed |

*Assumed to be the same as for 28ºC based on Fortuna et. al 2015 [9].

**Reference list Supplementary File 2**

1. Bowman C, Gumel AB, Van Den Driessche P, Wu J, Zhu H. A mathematical model for assessing control strategies against West Nile virus. Bull Math Biol. 2005;67:1107–33.

2. Pawelek KA, Niehaus P, Salmeron C, Hager EJ, Hunt GJ. Modeling dynamics of *Culex pipiens* complex populations and assessing abatement strategies for West Nile virus. Wang T, editor. PLoS One. 2014;9:e108452.

3. Blayneh KW, Gumel AB, Lenhart S, Clayton T. Backward bifurcation and optimal control in transmission dynamics of West Nile virus. Bull Math Biol. 2010;72:1006–28.

4. Nicholas Komar. West Nile virus: Epidemiology and ecology in North America. Adv Virus Res. Elsevier Inc.; 2003;61:185–234.

5. Laperriere V, Brugger K, Rubel F. Simulation of the seasonal cycles of bird, equine and human West Nile virus cases. Prev Vet Med. Elsevier B.V.; 2011;98:99–110.

6. Rubel F, Brugger K, Hantel M, Chvala-Mannsberger S, Bakonyi T, Weissenböck H, et al. Explaining Usutu virus dynamics in Austria: Model development and calibration. Prev Vet Med. 2008;85:166–86.

7. Esteva L, Yang HM. Assessing the effects of temperature and dengue virus load on dengue transmission. J Biol Syst. 2015;23:527-54.

8. Vogels CBF, Fros JJ, Göertz GP, Pijlman GP, Koenraadt CJM. Vector competence of northern European *Culex* pipiens biotypes and hybrids for West Nile virus is differentially affected by temperature. Parasites and Vectors. Parasites & Vectors; 2016;9:393.

9. Fortuna C, Remoli ME, Di Luca M, Severini F, Toma L, Benedetti E, et al. Experimental studies on comparison of the vector competence of four Italian *Culex pipiens* populations for West Nile virus. Parasit Vectors. Parasites & Vectors; 2015;8:463.

10. Wonham MJ, De-Camino-Beck T, Lewis MA. An epidemiological model for West Nile virus: Invasion analysis and control applications. Proc R Soc B Biol Sci. 2004;271:501–7.
