# Supplementary material for: The potential role of the Asian bush mosquito Aedes japonicus as spillover vector for West Nile virus in the Netherlands
Source: Parasit Vectors. 2024 Jun 17;17:262. doi: 10.1186/s13071-024-06279-5 (PMC11181672; doi:10.1186/s13071-024-06279-5)
Supplement: Supplementary file 3 — Additional file 3: Text S3. Estimation of biting rate and transmission probabilities. [file 13071_2024_6279_MOESM3_ESM.docx]

**Supplementary File 3:**

**Text S3:** Estimation of biting rate and transmission probabilities.

The competence of a mosquito is not only a function of how likely a pathogen is transmitted to a host, but also the rate at which the vector comes into contact with susceptible hosts. We interpret this vector biting rate as the inverse of the gonotrophic cycle duration. For *Cx. pipiens* the average is 5.54 ± 1.73 days [1]. We could not find estimates of the gonotrophic cycle for *Ae. japonicus*, so data on *Ae. albopictus* were used to inform the biting rate of *Ae. japonicus*, under the assumption that they have similar behaviour. Delatte *et al*. [2] estimated the average duration of gonotrophic cycle for *Ae. albopictus* for the temperature levels of 20, 25, and 30°C as 8.1, 4.5, and 3.5 days, respectively. We take the mean of the first two as a reasonable estimate for a temperature of 22°C (6.3 days) and the mean of the last two for a temperature of 28°C (4 days). Based on these assumptions, the biting rate of *Ae. japonicus* then becomes 0.16 /day and 0.25 / day, for 22 and 28°C respectively.

The transmission probability, denoted as *p_i_*, is defined as the probability that the virus is transmitted upon a mosquito bite. Six different transmission probabilities can be distinguished in our model: *Ae. japonicus* to bird, *Ae. japonicus* to human, *Cx. pipiens* to bird, *Cx. pipiens* to human, bird to *Ae. japonicus*, and bird to *Cx. pipiens*. The bird-to-mosquito transmission probabilities differ by temperature and isolate. Unless stated otherwise, we assume equal transmission probabilities upon biting for *Cx. pipiens* and *Ae. japonicus*.

The value of the transmission probabilities from bird to each mosquito species are based on the infection rate results presented in Table 1. The transmission probability as used in the model is equal to the transmission efficiency in Table 1. Because these transmission efficiencies were measured in mosquitoes that had all taken an infectious blood meal and were tested 14 days after this blood meal, we also explicitly model the biting rate and the extrinsic incubation rate.

The value of the transmission probability from mosquito to host was assumed to be the same for all combinations of mosquito species and host species and was estimated at 0.88 [3].

In the experiments that were carried out, some mosquitoes likely had not fully completed the extrinsic incubation period after 14 days. This could lead to underestimating their transmission potential from the data. We corrected for this in the following way. The measurements were done after 14 days. The rate at which virus incubates in mosquitoes is assumed to be exponential. So, if the incubation time follows an exponential distribution, after 14 days 1-*e^-λt^**100% of mosquitoes have completed the incubation period, where *λ* is the incubation rate and *t* is the time (14 days). The transmission probabilities are then multiplied by 1+*e^-λt^*. For *Cx. pipiens*, for example, the incubation rate at 22 ºC is 0.11 [25], so after 14 days approximately 79% have completed the EIP. This means that the 21% that are not yet infectious are not being accounted in the estimation of the transmission probability (0.04), so we multiply that probability by 1.21 giving 0.048.

Additionally, the mosquitoes still incubating at 14 days may not survive the incubation period after 14 days. This could lead to an overestimation of the transmission probability in the correction for incubation period. To account for this mortality, we multiply the correction factor described above by the probability for mosquitoes to survive longer than 14 days. This probability follows an exponential distribution where μ is the death rate of mosquitoes. The probability to survive after 14 days is *e^-μt^*, where *μ* is the mortality rate of the mosquitoes and t=14 days. For *Cx. pipiens*, for example, the death rate at 22º C is 0.1677, so the probability to survive more than 14 days is 0.10. Thus, the transmission probabilities are multiplied by the proportion of mosquitoes still incubating times the probability to survive longer than 14 days: the correction factor therefore is 1+*e^-λt^ e^-μt^*.

**Reference List Supplementary File 3**

1. Faraj C, Elkohli M, Lyagoubi M. The gonotrophic cycle of *Culex pipiens* (Diptera: *Culicidae*), West Nile virus potential vector, in Morocco: evaluation of its duration in laboratory. Bull la Soc Pathol Exot. 2006;99:119–21.

2. Delatte H, Gimonneau G, Triboire A, Fontenille D. Influence of temperature on immature development, survival, longevity, fecundity, and gonotrophic cycles of *Aedes albopictus,* vector of chikungunya and dengue in the Indian Ocean. J Med Entomol. 2009;46:33–41.

3. Wonham MJ, De-Camino-Beck T, Lewis MA. An epidemiological model for West Nile virus: Invasion analysis and control applications. Proc R Soc B Biol Sci. 2004;271:501–7.
