# Supplementary material for: The potential role of the Asian bush mosquito Aedes japonicus as spillover vector for West Nile virus in the Netherlands
Source: Parasit Vectors. 2024 Jun 17;17:262. doi: 10.1186/s13071-024-06279-5 (PMC11181672; doi:10.1186/s13071-024-06279-5)
Supplement: Supplementary file 4 — Additional file 4: Text S4 Derivation of the basic reproduction number. [file 13071_2024_6279_MOESM4_ESM.docx]

**Supplementary File 4:**

**Text S4:** Derivation of the basic reproduction number.

The basic reproduction number (*R_0_*) was derived through the next-generation matrix method, which we describe in detail in this section.

To find *R_0_* we select the states-at-infection of the above system and write the matrices of transmissions, ***T***, and transitions, ***Σ***, given by


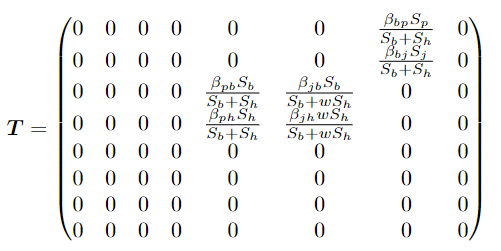


and


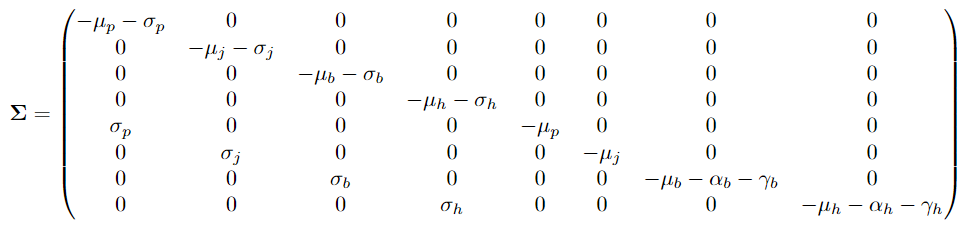


Matrix ***T*** contains four rows consisting only of zeros, so we define an intermediate matrix ***E*** as in [1] as


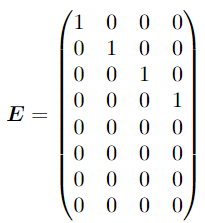


and obtain the next-generation matrix ***K*** by calculating ***E' K E*** which then becomes


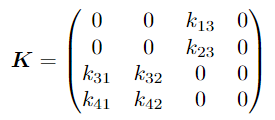


with


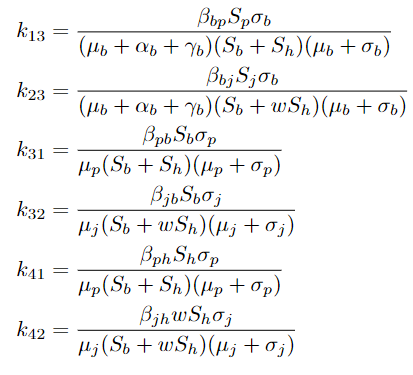


where the species are evaluated at their disease-free equilibrium abundances given by

$$S_{p}^{*}=\frac{\Delta_{p}\left( T \right)}{\mu_{p}\left( T \right)}, S_{j}^{*}=\frac{\Delta_{j}\left( T \right)}{\mu_{j}\left( T \right)}, S_{b}^{*}=\frac{\Delta_{b}}{\mu_{b}}, S_{h}^{*}=\frac{\Delta_{h}}{\mu_{h}}$$

The dominant eigenvalue of this matrix evaluated at this disease-free equilibrium corresponds to *R_0_*, and is given by

$$R_{0}=\sqrt{k_{13}k_{31}+k_{23}k_{32}}=$$

$$=\sqrt{\frac{S_{b}^{*}\sigma_{b}}{\left( \mu_{b}+\sigma_{b} \right)\left( \mu_{b}+\alpha_{b}+\gamma_{b} \right)}\left( \frac{b_{p}\left( T \right)p_{pb}\left( T \right)S_{p}^{*}\sigma_{p}\left( T \right)}{\mu_{p}\left( T \right)\left( S_{b}^{*}+S_{h}^{*} \right)^{2}\left( \mu_{p}\left( T \right)+\sigma_{p}\left( T \right) \right)}+\frac{b_{j}\left( T \right)p_{jb}\left( T \right)S_{j}^{*}\sigma_{j}\left( T \right)}{\mu_{j}\left( T \right)\left( S_{b}^{*}+wS_{h}^{*} \right)^{2}\left( \mu_{j}\left( T \right)+\sigma_{j}\left( T \right) \right)} \right)}$$

**Reference list Supplementary File 4:**

1. Diekmann O, Heesterbeek JAP, Roberts MG. The construction of next-generation matrices for compartmental epidemic models. J R Soc Interface. 2010;7:873–85.
