# Supplementary material for: The potential role of the Asian bush mosquito Aedes japonicus as spillover vector for West Nile virus in the Netherlands
Source: Parasit Vectors. 2024 Jun 17;17:262. doi: 10.1186/s13071-024-06279-5 (PMC11181672; doi:10.1186/s13071-024-06279-5)
Supplement: Supplementary file 5 — Additional file 5: Text and Figure S5 Elasticity of the basic reproduction number. [file 13071_2024_6279_MOESM5_ESM.docx]

**Supplementary File 5:**

**Text S5:** Elasticity of the basic reproduction number.

To test the impact of the model parameters on the basic reproduction number, *R_0_*, an elasticity analysis was carried out. This provides information on which parameter changes contribute the most to *R_0_*. We were particularly interested in the parameters whose value was not known, and therefore assumed to have a pre-specified value in our simulations: *w*, *σ_j_*, *p_jb_*, and *p_jh_* (the latter two are expressed as transmission rates which include the biting rate *b* and the transmission probabilities *p_ij_*, i.e., *β_jb_* = *b_j_ p_jb_*, and *β_jh_* = *b_j_ p_jh_*). **Figure S5** shows the elasticity of *R_0_* for the parameters only in the scenario of the Dutch isolate at 22 ºC, since the results for the remaining scenarios were similar. The values for the parameters *w*, *σ_j_*, and *p_jh_*, have a relatively small impact on the value of *R_0_*, while those related to *Ae. japonicus, p_jb_* , *p_bj_*, and *b_j_*, contribute to the largest increase in *R_0_*.


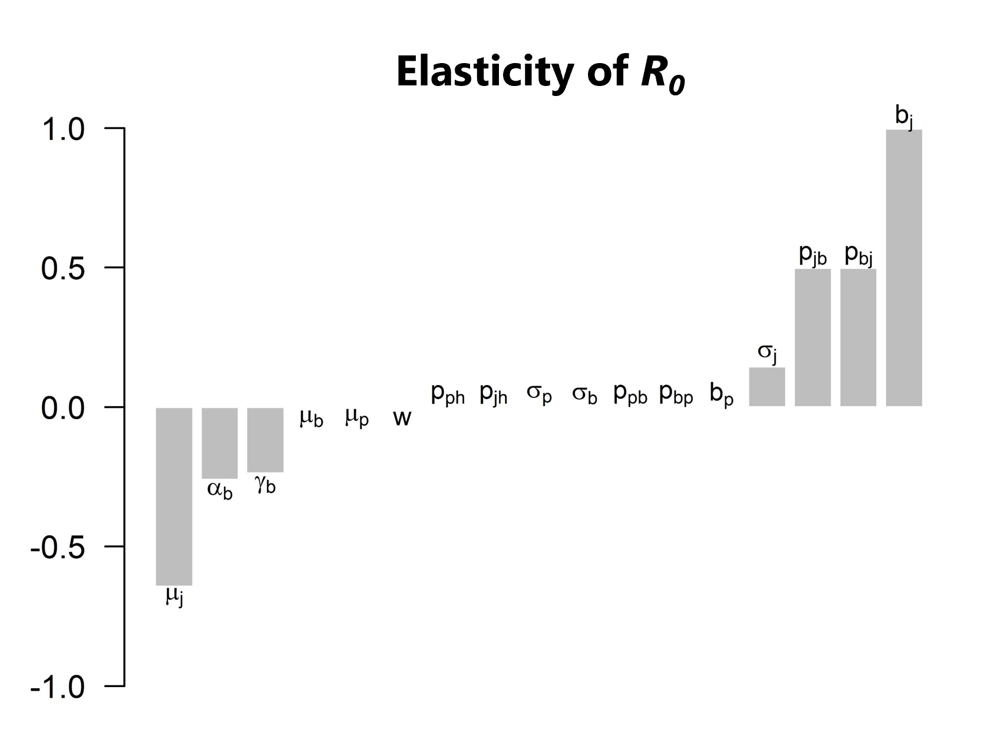


**Figure S5**: Elasticity of R_0_ to the model parameters at the disease-free equilibrium. The parameter values used are shown in Supplementary table S2. A positive value is interpreted as an increase in R0 while a negative value corresponds to a decrease in R_0_.
